# RNA-seq analysis identifies key genes enhancing hoof strength to withstand barefoot racing in Standardbred trotters

**DOI:** 10.1186/s12864-025-11814-4

**Published:** 2025-08-18

**Authors:** Doreen Schwochow, Asmaa Alameddine, Ellinor Spörndly-Nees, Mathilde Montigny, Rakan Naboulsi, Anna Jansson, Adnan Niazi, Gabriella Lindgren

**Affiliations:** 1 Department of Animal Biosciences, Swedish University of Agricultural Sciences, Uppsala, Sweden; 2 Division of Gene Technology, Science of Life Laboratory, Royal Institute of Technology, Stockholm, Sweden; 3 Department of Pathology and Wildlife Diseases, Swedish Veterinary Agency, Uppsala, Sweden; 4 Childhood Cancer Research Unit, Department of Women’s and Children’s Health, Karolinska Institute, Stockholm, Sweden; 5 Center for Animal Breeding and Genetics, Department of Biosystems, KU Leuven, Louvain, Belgium

**Keywords:** Horse, Hoof strength, Barefoot racing, RNA-seq, Vasoconstriction, Metal homeostasis, Ceramides, Keratinization, Inflammatory memory

## Abstract

**Background:**

Racing without protective shoes is common in the Swedish harness racing industry, as it can enhance horses’ performance on the track. Trainers typically decide whether a horse will race barefoot based on practical experience rather than objective measures. However, this practice can sometimes lead to excessive hoof wear, posing potential welfare concerns for racing horses. Gene expression differences may help reveal the underlying genetic mechanisms associated with different phenotypic traits. To explore an objective measure for assessing which horses are best suited for barefoot racing, we conducted a polyA-selected RNA-seq experiment on tissue from the growth zone at the coronary band of the hoof. This experiment compared tissues from Standardbred trotters capable of repeatedly racing barefoot without injury (n = 11) to those that could not (n = 7). By combining stringent phenotyping with racing records and trainer interviews, we aimed to elucidate the biological factors related to hoof strength in barefoot racing, focusing on differential abundant genes.

**Results:**

The RNA-seq analysis identified five significantly downregulated genes in horses capable of competing barefoot across consecutive races. These genes are associated with various biological processes relevant for hoof strength: *ACCS*, *IRX2* and *TRAPPAC6A* contribute to enhancing the structural integrity of the hoof; *MT2A* regulates its metal homeostasis and *SLC35F3* likely influences local vasoconstriction in the hoof. These gene findings suggest a coordinated genetic basis for structural reinforcement and physiological support of the hoof, which may be critical for sustaining performance under barefoot conditions.

**Conclusion:**

Our findings suggest that the ability of Standardbred trotters to race barefoot in consecutive events is reflected in distinct gene expression patterns, underscoring a genetic basis for hoof strength. This supports further genome-wide scans aimed at identifying genetic markers for hoof durability in these horses. The focused design of our study– comparing horses that could consistently race barefoot with those that could not– enabled us to isolate a select group of genes involved in diverse aspects of hoof biology essential for quality and resilience of horse hooves. This insight could ultimately be applied to augment both the performance and wellbeing of equine athletes across disciplines.

**Supplementary Information:**

The online version contains supplementary material available at 10.1186/s12864-025-11814-4.

## Background

**Fig. 1:**
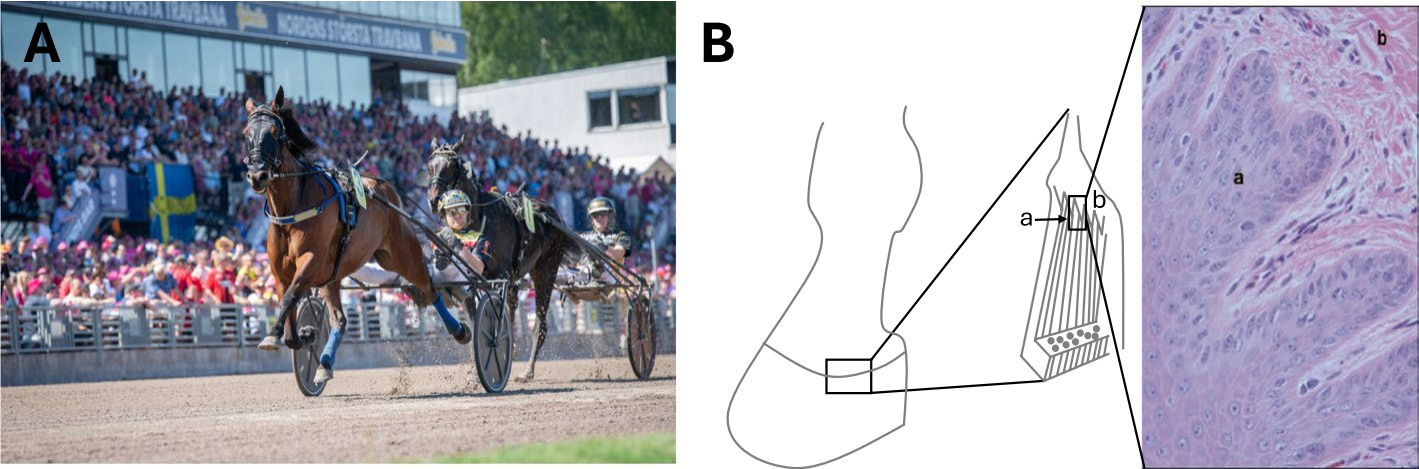
Sampling of hooves from horses competing in Swedish harness racing. **A** Barefoot harness racing Standardbred horse. (Horse: Krakas; driver: Mats E. Djuse; Photo credit: Mathilda Persson). **B** Schematic illustration of tissue origin lateral side of right hind hoof at the coronary band. The tissue was collected in the growth zoon at the proximal region of the hoof wall in the coronary groove were the tubules that form the wall grow. The samples were taken at (**a**) the proximal part of the coronary papillae, where the proliferation of the keratinocytes accrue (**b**) connective tissue, coronary corium. Stain: hematoxylin eosin

The equestrian adage "No foot, no horse" underscores the vital importance of healthy and robust hooves in horses. The condition of a horse's hooves profoundly impacts its movement and susceptibility to injuries, as disruptions in hoof homeostasis can have far-reaching consequences [1]. To counteract potential damage, shoeing is a common practice aimed at protecting the hoof from wear, particularly during strenuous exercise or challenging footing. While horseshoes offer protective benefits, they also alter the hoof's natural biomechanics, including blood circulation and expansion during movement, and add extra weight [2]. In Swedish harness racing (Fig. 1A), it is widely believed that racing barefoot (without shoes) allows horses to run faster, increasing their chances of victory. This belief was supported by a study that confirmed barefoot racing improves speed but also revealed a heightened risk of galloping, which can lead to disqualification [3]. Interestingly, this risk can be mitigated by shoeing only the hind hooves, as research on both horses and cattle suggests that hind hooves or claws are more prone to damage than their front counterparts [4, 5].

A common approach in Swedish harness racing is to remove horseshoes shortly before the race. This strategy minimizes daily wear and tear while preserving hoof integrity for the demands of racing. However, the decision to race barefoot often relies on the trainer's experience and trial-and-error, as there are no objective measures of hoof quality. This lack of standardization poses potential welfare concerns for harness racing horses.

The mechanical properties and durability of the hoof are heavily influenced by its composition. Compared to horse hooves, the structure and health of hoof horn in dairy cattle have been more extensively studied, given that lameness is a major economic and welfare issue in the dairy industry [6]. In cattle, hoof horn condition has been primarily linked to diet (e.g., [6–8]) and management practices like trimming [9–11], but hormonal and genetic factors also play a role [12, 13]. Adequate intake of fatty acids, minerals, vitamins, and amino acids—particularly sulfur-containing amino acids—is essential for producing a strong hoof capsule [7]. Notably, sulfur concentrations are highest in the parts of the hoof that bear the greatest weight, emphasizing its role in structural integrity [14].

Deficiencies in trace minerals such as zinc, copper, and manganese are associated with lameness in dairy cows, leading to reduced hoof hardness, fragmentation, and disorganized collagen fibers [15]. Similar patterns are beginning to emerge in horse hooves. Recent research indicated that horses competing frequently in barefoot harness races exhibited higher concentrations of cysteine, arginine, sulfur, and copper compared to their less often barefoot racing competitors [5]. These differences were attributed to changes in keratin composition, a protein derived from amino acids like cysteine and arginine. Sulfur facilitates the formation of strong disulfide bonds, which tightly link keratin filaments and enhance the durability of hoof fibers.

Additionally, keratin undergoes post-translational modifications that alter its amino acid profile, a phenomenon observed between more and less frequently competing horses [5]. Variations in copper levels were also noted, as copper is essential for enzymes like thiol oxidase, which catalyzes the formation of disulfide bonds in keratin filaments [16]. These findings underscore the intricate relationship between hoof composition, performance demands, and the nutritional and biochemical factors that sustain hoof health.

Keratinization and cornification are complex processes driving the formation of skin and its appendages, including nails and hooves, through keratinocyte differentiation and programmed cell death. This process, essential for creating tough, protective structures, is regulated by pathways like Wnt and Notch, transcription factors such as p63, and epidermal growth factors. Studies in mammals, including *Msx2* and *Foxn1* mutant mice, reveal that disruptions in keratin production result in weaker nails and poorly differentiated keratinocytes [17, 18]. Keratins are encoded in the genome and are produced in a temporal and spatial manner [19]. These proteins, together with other protein such as filaggrin, are polymerized and cross-linked by keratinocyte-specific transglutaminase and sulfuryl oxidase in a calcium-dependent manner so that keratin filaments become very tightly packed and covalently bound to the cell envelope [16, 20].

In horses, the genetics of hoof strength remain understudied, though breed-based differences in hoof composition suggest a genetic role i.e., [21–23]. The Baicha Iron Hoof horse, a horse breed adapted to rocky terrain, may provide insights. Recent findings have identified selection signatures associated with hoof resilience in this breed [24]. Given the limited understanding of genetic influences on hoof health and the welfare implications of barefoot racing, further research is needed.

The purpose of this study was therefore to compare the gene expression from the growth zone of the coronary band in hind hooves of Swedish Standardbred trotters that were able to race barefoot in successive races (barefoot—B) to those horses that were not capable of it, but instead were wearing shoes during competitions more often (non-barefoot—NB). The exact same set of horses were previously investigated for their hoof content [5], making it possible to compare the data on composition of the hoof and a potential underlying genetic basis. We hypothesized that those two groups a priori express a distinct set of genes involved in pathways affecting either the hoof composition and strength or the suppliance of the hoof with essential nutrients, and that these genes could pave the road to better understand the genetic basis of hoof strength in general and in racing horses in particular.

## Material and methods

### Animals and selection criteria

Standardbred trotters aged between 5–21 years with a minimum of eight races during their career were included in the study, along with information on sex (Table 1; Fig. 1A). Horses were only considered to regularly race barefoot (B) if they raced barefoot three times within a one-month period (31 days) at least once in their racing career. Horses that were considered racing shod/not barefoot (NB) could occasionally have raced barefoot, but then with a minimum of 45 days between shoeless racing. The trainer of those horses had to confirm that the horses cannot race barefoot often as a result of too heavy wear and tear of the hind hooves. Data on racing condition (barefoot or not) and trainers were acquired from the Swedish Trotting association. Hoof material was obtained from horses that were culled for other reasons than this study. 11 of the collected horses (12 ± 6 years) were categorized in the B group, while 7 horses were part of the NB group (16 ± 5 years).

**Table 1:** Horses used for RNA-seq and qPCR validation

Sample ID	Group	Sex	Age (years)	Time since last race (months)	Used for
H1	NB	M	10	0	RNA-seq, qPCR
H2	NB	F	20	156	RNA-seq, qPCR
H3	B	F	10.5	73	RNA-seq, qPCR
H4	B	F	20	133	RNA-seq, qPCR
H5	NB	F	16.5	83	RNA-seq, qPCR
H6	B	M	11.5	61	RNA-seq, qPCR
H7	B	F	12.5	106	RNA-seq, qPCR
H10	B	F	5.5	4	RNA-seq, qPCR
H12	B	F	12.5	5	RNA-seq, qPCR
H14	B	F	16	126	RNA-seq, qPCR
H15	NB	F	17	98	RNA-seq, qPCR
H16	NB	F	11	75	RNA-seq, qPCR
H17	B	F	11	18	RNA-seq, qPCR
H20	B	M	5	1	RNA-seq, qPCR
H22	B	M	6	20	RNA-seq, qPCR
H23	B	F	13	71	RNA-seq, qPCR
H24	NB	M	7.7	1	RNA-seq
H27	NB	F	20.5	177	RNA-seq

*M* male, *F* female, *NB* not barefoot, *B* barefoot in successive races

### Sample collection

In line with the 3Rs principles (Replacement, Reduction, and Refinement), tissues for this study were collected from horses already euthanized at a licensed abattoir in Sweden for reasons unrelated to the study. None of the horses was put down due to acute illness or direct hoof related conditions. The right and left hind hooves were collected immediately after culling and transported on ice to the laboratory. The hooves were cut with a band saw in four pieces by a sagittal section (caudo-cranial direction) and a transverse section (latero-medial direction). The sample for the genetical analysis were obtained from the lateral side at the coronary band. A piece (2 mm × 2 mm) was cut out at the coronary groove where the growth of the hoof walls tubular structure takes place (location ´a´ in Fig. 1B). Samples were stored at −80 °C for further processing.

### RNA preparation

RNA was extracted using the RNeasy Fibrous Tissue Kit (Qiagen Inc., Valencia, USA) following the manufactures recommendation for fibrous tissue. 10–20 mg of tissue from the coronary band of the hoof were homogenized in RTL buffer using a Precellys Evolution instrument (Bertin Instruments, Montigny-le-Bretonneux, France) for 3 × 30 s at 6500 rpm. RNA quality and quantity was measured using NanoDrop 1000 Spectrophotometer (Thermo Scientific, USA) as well as on a 4150 TapeStation System (Agilent Technologies, Inc, USA) using High Sensitivity RNA ScreenTape® (Agilent Technologies, Inc, USA) and TapeStation Analysis Software 3.2 (Agilent Technologies, Inc, USA).

### Library preparation

Library preparation was performed by the SNP&SEQ Technology Platform, a national unit within the National Genomics Infrastructure (NGI), hosted by Science for Life Laboratory in Uppsala, Sweden. Only RNA samples with RIN values ≥ 7 were used for library preparation. In short, sequencing libraries were prepared from 500 ng total RNA using polyA selection and the TruSeq stranded mRNA library preparation kit (Illumina, San Diego, CA, USA). Unique dual indexes were used (wasmina). The library preparation was performed according to the manufacturer’s protocol (#1,000,000,040,498). The quality of the libraries was evaluated using the Fragment Analyzer (Advanced Analytical Technologies, Inc., Santa Clara, CA, USA) and the DNF-910 dsDNA kit. The adapter-ligated fragments were quantified by qPCR using the library quantification kit for Illumina (KAPA Biosystems, Wilmington, MA, USA) on a CFX384 Touch instrument (Bio-Rad, Hercules, CA, USA) before cluster generation and sequencing. Sequencing was carried out on an Illumina NovaSeq 6000 instrument (NSCS v 1.7.0/RTA v 3.4.4) according to the manufacturer’s instructions (Illumina).

### RNA-seq analysis

Demultiplexing and conversion to FASTQ format were performed using the bcl2fastq2 (2.20.0.422) software provided by Illumina [25]. Additional statistics on sequencing quality were compiled with an in-house script from the FASTQ files, RTA, and BCL2FASTQ2 output files. The RNA-seq data were analyzed using the best practice pipeline nf-core/rnaseq v1.4.2 [26]. Briefly, raw reads were trimmed for low quality sequences, and adaptor sequences were removed using Trim Galore v0.6.6 [27]. Quality filtered reads were mapped against the reference genome of *Equus caballus* (EquCab3.0 v104) using STAR aligner v 2.6.1 (PMID: 23,104,886) to assess the mapping percentage of the RNAseq data. Gene expression was quantified from transcriptome of *Equus caballus* using salmon v1.4.0 [28]. Normalization of gene counts and differential expression (DE) analysis of the quantified genes was performed with the R package DESeq2 version 1.32.2 [29]. Two models were fitted to assess the effect of different covariates on gene expression. The first model included ‘age’ and ‘sex’ as covariates, while the second model included ‘age,’ ‘sex’ and ‘time since last race’. After DE analysis, the resulting *p*-values were adjusted for multiple testing using the Benjamini–Hochberg procedure [30]. DEGs with adjusted *p*-values < 0.05 and base mean expression > 50 were considered significant.

### cDNA synthesis and qPCR

The majority of RNA samples used for RNA-seq could also be used for validation of differentially expressed genes (Table 1). If sufficient RNA yield was obtained, 1 μg RNA was used to reverse transcribe using the High-Capacity cDNA Reverse Transcription kit (Applied Biosystems™, MA, USA) following the manufacture’s recommendation (including RNA inhibitors). The cDNA concentration was measured using a Qubit fluorometer (Invitrogen Life Technologies, MA, USA) following the standard protocol for ssDNA assay measurements.

Primers were designed using the Primer3Plus software [31]. The primers were tested for their PCR efficiency in a four-point standard curve and their specificity was evaluated using melting curve analysis. Primer sequences are provided in S1 Table. In order to validate the gene expression of the differentially expressed genes obtained from the RNA-seq experiment, 1 ng cDNA (*IRX2*) or 10 ng cDNA were used with 1 μM of each primer using a SYBR^TM^Green Real-Time PCR Master Mix (Life Technologies, Warrington, United Kingdom) on a StepOnePlusTM Real-Time PCR System (Applied Biosystems™, MA, USA) with standard PCR conditions using StepOne™ Software v.2.3 (Applied Biosystems™, MA, USA). The obtained C_t_ values were analyzed using the ΔΔCt method. Relative expression of the target gene was normalized using β-actin as housekeeping genes. All samples were run as triplicates. Unpaired Student’s t-test was used to assess statistically significant differences in differential expression of target genes in the different horse groups.

## Results

### RNA-seq

RNA sequencing generated 55–380 million reads per sample. Approximately 74%–84% read pairs per sample mapped uniquely to the genome. Principal component analysis using all expressed genes showed no overall difference between shod and barefoot horses (S Fig. 1). Differential expression analysis identified five significantly differentially expressed genes (DEGs) (*1-aminocyclopropane-1-carboxylate synthase homolog*; *ACCS; solute carrier family 35 member F3*; *SLC35F3; trafficking protein particle complex subunit 6 A; TRAPPC6A; Iroquois homeobox 2*; *IRX2* and *metallothionein 2A*; *MT2A*) with an adjusted *p*-value < 0.05 and a base mean expression > 50 (Table 2). In the B group, all genes were under expressed in comparison to the NB group. The five genes are involved in different cellular processes such as membrane transport (*SLC35F3*) or shuttling vesicles between the cell compartments (*TRAPPC6A*) or participate in metabolic processes (*ACCS*), activate gene transcription (*IRX2*) or bind to various components such as ions, lipids etc. in the cell (*MT2A*). All expressed genes for both groups are displayed in S2 Table. Three of these genes; *ACCS, SLC35F3* and *TRAPPC6A* were also found significantly differentially expressed (padj < 0.05) when ‘time since last race’ was included as an additional covariate in the model. No additional genes were identified in the extended model.

**Table 2:** List of differentially expressed genes in hooves of horses able to race barefoot (B) in successive races by RNA-seq

Ensemble accession number	Gene symbol	pval	padj	FC	Function
ENSECAG00000013484	*ACCS*^a^	2.22E-08	0.0002	−1.849	Catalyzes the deamination of L-vinylglycine
ENSECAG00000007284	*SLC35F3*^a^	1.05E-06	0.0044	−1.589	Mediates thiamine transport
ENSECAG00000018946	*TRAPPC6A*	6.25E-06	0.0148	−1.587	Vesicle transport to *cis*-Golgi membrane
ENSECAG00000015758	*IRX2*^a^	7.19E-06	0.0148	−2.661	Transcription factor involved in various cellular processes such as proliferation, differentiation and migration
ENSECAG00000038313	*MT2A*	7.65E-06	0.0148	−1.960	Binds heavy metal ions, altering intracellular concentration of heavy metals

Genes included in this list had a padj ≤ 0.05 and base mean expression > 50

*pval* p value, *padj* p value adjusted, *FC* fold change

^a^validated through qPCR

### qPCR validation of DEG

**Fig. 2:**
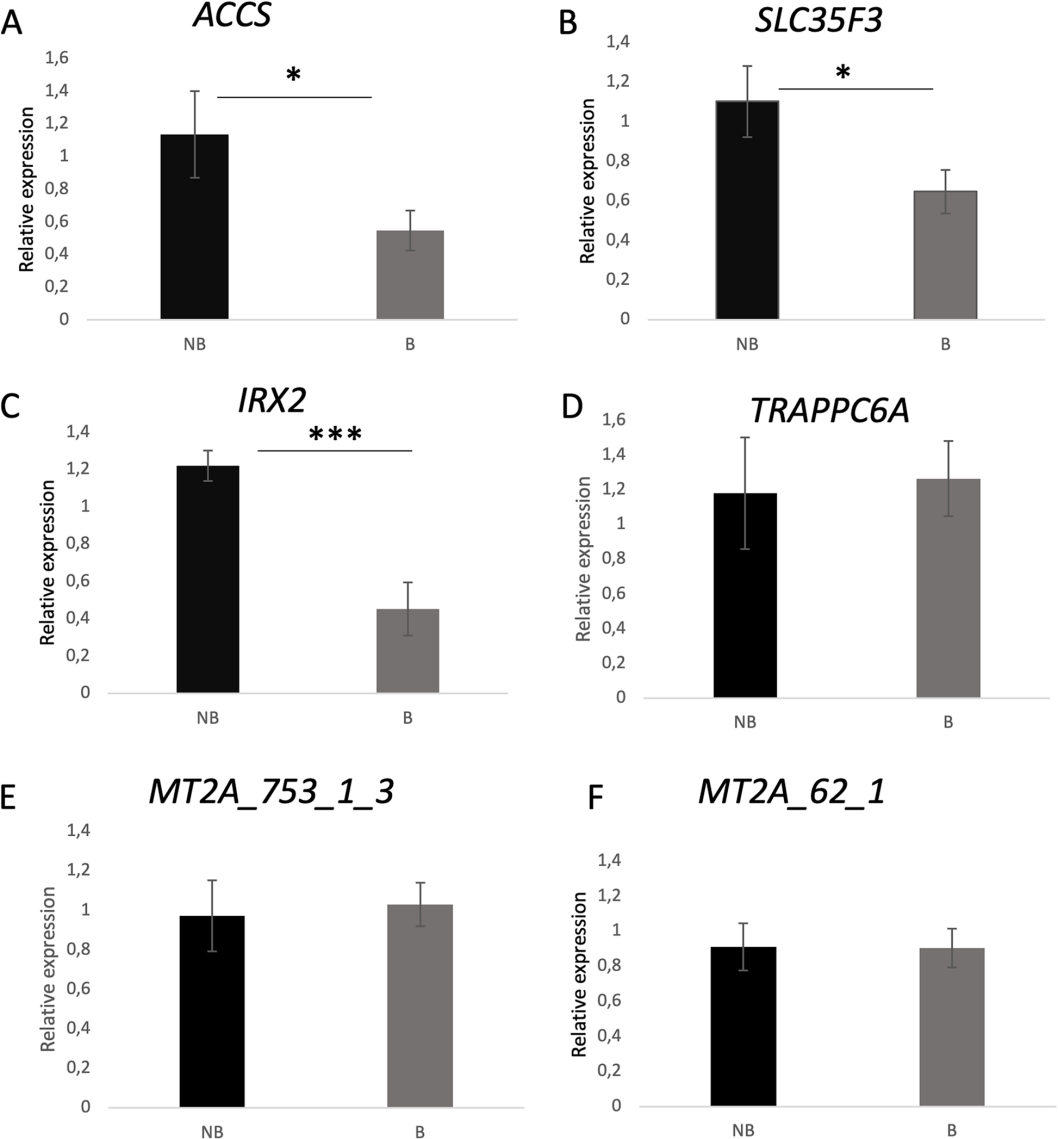
qPCR validation of RNA-seq data from hooves of horses racing with or without shoes. Relative gene expression of genes, in the hoof lamina of horses frequently racing without shoes (B; N = 9–11) or with shoes (NB; N = 4–5). For *MT2A* two different primer pairs were used. Expression data was normalized using β-actin (Student’s t-test; * *P* < 0.05; ** *p* < 0.005; ***p < 0.0005)

To validate the RNA-seq data, qPCRs were performed for all five genes that showed significant differential expression (adjusted *p*-value < 0.05 and a base mean expression > 50); (Table 2) between B and NB horses: *ACCS*, *SLC35F3*, *TRAPPC6A, IRX2* and *MT2A*. The qPCRs confirmed that *ACCS* (Average = 0,54 ± 0.1; Student’s t-test* P* = 0.04), *SLC35F3* (Average = 0,64 ± 0.1; Student’s t-test* P* = 0.04) and *IRX2* (Average = 0,45 ± 0.1; Student’s t-test* P* = 0.0004) are indeed under expressed in horses that were able to race barefoot more often (Fig. 2). The average relative expression level for *MT2A* and *TRAPPC6A* did not differ substantially between the two groups (Average = 0,9 ± 0.1 for primer pair MT2A_62_1_1/Average = 1,0 ± 0.1 for primer pair MT2A_753_1_3 and Average = 1,3 ± 0.2 for *TRAPPC6A*) and did not reach statistical significance (*P* = 0.9/*P* = 0.8 and *P* = 0.3 respectively).

## Discussion

**Fig. 3:**
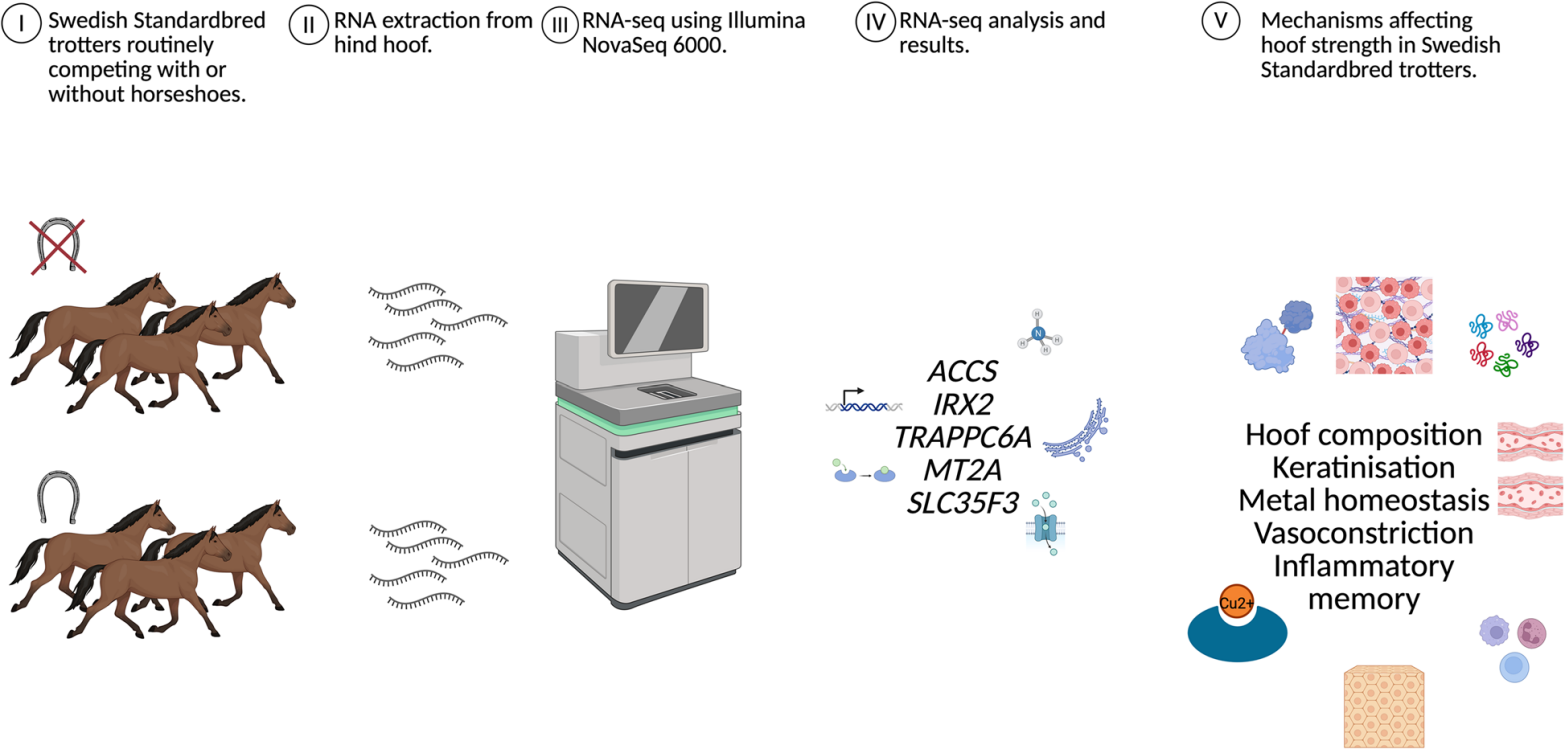
Graphical abstract illustrating both experimental design and results. DEG = differentially expressed genes. Figure was crafted in BioRender.com

Using RNA-seq, we identified five genes significantly differentially expressed between horses that consistently raced barefoot and those that could not. This focused study design—comparing horses of the same breed under similar training conditions, most of them without recent barefoot racing exposure—allowed us to pinpoint a small set of genes linked to hoof resilience rather than immediate post-race effects (Fig. 3). Given the similarities between the groups (frequently racing barefoot vs. occasional racing barefoot), this stringent phenotyping resulted in minimal differences, which, while limiting the number of genes detected, allowed for a focused analysis on a select group of genes linked to essential aspects of hoof biology, quality, and resilience in horses adapted to barefoot racing.

### *ACCS*, *IRX2* and *TRAPPC6A* may lay the molecular basis for a resilient hoof composition in barefoot racing horses

Our data shows that horses capable of racing barefoot in successive competitions exhibit lower expression of *1-aminocyclopropane-1-carboxylate synthase-like protein* (*ACCS*). ACCS enzymatic reaction releases ammonia from vinylglycine, so that overexpression of *ACCS* may lead to high ammonia concentrations, which can be detrimental to cells and tissues [32]. A 2009 study found, that increased ammonia levels correlated with reduced ceramide content in cow claw horn [33]. Ceramides, intracellular lipids, which are part of the lipid lamellar structure of nails [34], are crucial for maintaining skin hydration and hoof tissue flexibility [35, 36]. This suggests that lower ACCS activity could reduce ammonia levels, positively affecting hoof ceramide content and tissue homeostasis in barefoot racing horses.

It is interesting to note, that another gene, that is also under expressed in horses that can race barefoot more frequent, can be connected to the production of the ceramide layer during the keratinization process. *Trafficking protein particle subunit complex 6A* (*TRAPPC6A*), is a component of the trafficking protein complex of the Golgi membrane and recognized for its impact on skin biology through affecting pigmentation traits [37, 38]. The Golgi network is also impacting keratinization by producing lamellar bodies– secretory organelles, which transport among other components, acylceramides [39]. The content of the lamellar bodies is fusing with the keratinocyte membrane through exocytosis and gradually increases its acylceramide content [39], which is later tightly connecting neighboring corneocytes [36] increasing tissue stability and improving the moisture content in the hoof.

Our gene expression analysis detected yet another gene implicated in skin appendage biology. *Iroquois homeobox 2* (*IRX2*), is coding for a transcription factor, which is under expressed in horses that were able to race barefoot more often. *IRX2* has important roles during embryonal development [40–42] and cell cycle regulation in adult tissues [43, 44]. Its role in nail and hoof formation is less well established, but it has been shown to be highly expressed during skin keratinocyte differentiation and—along with *IRX4* and *IRX5*—necessary for proper cell cycle progression and maintenance in basal keratinocytes [45]. Knockdown of *IRX2* (along with *IRX4* and *IRX5*) disrupts normal keratinocyte differentiation and alters expression of genes involved in epidermal development, keratinization, cell–cell adhesion and cornification, which very likely extends to nails and hooves through shared cellular mechanisms. As keratinocytes differentiate, they change the types of keratins they express, which affects structural support and resilience of the different layers formed [46–48]. We found previously that horses able to race barefoot in consecutive races tended to have higher nitrogen levels and significantly higher levels of arginine [5], a finding that is not reflected in differential expression of genes concerning amino acid metabolism or other enzymes involved in similar pathways, but could suggest differences in keratin composition as a result of differential regulation of *IRX2*. This might provide some horses with an advantage to withstand the wear and tear from barefoot racing better or/and respond more efficiently to wounds and injuries.

Overall, these novel molecular insights highlight the genetic and biochemical factors contributing to hoof strength and integrity in barefoot racing horses.

### *MT2A* is involved in metal and sulfur homeostasis in barefoot racing horses

Horses that race barefoot more frequently exhibit lower expression of a gene involved in metal homeostasis called *metallothionine* (*MT2A*, Table 2*)*. Copper is a crucial mineral for basic cell and tissue survival and metabolism, including protein synthesis and connective tissue formation processes [49–52]. In skin and hoof it is essential for activating thiol oxidase, an enzyme which catalyzes bond formations between keratin structures.

Studies on cattle show that, high dietary copper and zinc content may delay the onset and decrease laminitis severity [53], while deficiencies lead to claw disease [54], with supplementation reducing lameness. Evidence of copper's impact on hoof strength in horses and donkeys is inconclusive [55, 56], with some studies even suggesting that high copper content correlates with fragile hooves and poor hoof growth [5]. Our data shows lower expression of *MT2A,* which gene’s products bind to metal ions and thereby regulate metal ion concentration, suggesting low copper in live hoof tissue as recently observed in the horses that were able to race barefoot more often [5]. Alternatively, cellular copper might be high, with enzymes that reduce it being downregulated, making copper available for enzymes for keratin formation.

Not just copper is required for keratin bond formation, but also sulfur. Disulfide bonds impact the strength of the kreatin filaments and require the availability of sulfur, which is well known to impact nail strength in humans [57]. Horses, that could race barefoot in consecutive races showed a trend towards higher cysteine and higher sulfur concentration [5]. Those changes do not appear to be reflected in alternate expression of genes directly involved in keratin formation, amino acid or sulfur metabolism (S3 Table). However, MT2A does contain a high level of cysteine residues, which are rich in sulfur and play an important role in sulfur-based detoxification and redox regulation [58–60]. If the gene is downregulated in horses able to race barefoot in consecutive races, this could mean that more sulfur and cysteine is available for creating disulfide bonds and strengthen keratin filaments for greater hoof strength. This would not just align with the analyzed hoof content of the horses used in this study, but was confirmed by a set of studies, which associated higher sulfur and cysteine contents with stronger hooves and claws [5, 14, 61, 62].

A tight regulation of metal content in hoof-forming tissue is furthermore crucial, as enzymes requiring metal cofactors are involved in extracellular matrix (ECM) remodeling. The ECM is a complex network of macromolecules that provide structural and mechanical support to the cells of a tissue, facilitates intracellular signaling, is involved in regulating cell differentiation and homeostasis, aging as well as healing and various pathological aspects of tissues [63]. Matrix metalloproteinases (MMPs), zinc-dependent enzymes, cleave ECM macromolecules, affecting cell differentiation and homeostasis. Tight regulation of these enzymes is therefore essential for hoof health and durability, which is critical for barefoot racing. A study on Mongolian horses, known for their strong hooves, found selective sweeps near genes related to MMP2 activity, suggesting a genetic basis for durable hoof walls suitable for successive barefoot racing [24].

### *SLC35F3* and blood pressure regulation in barefoot racing horses

*SLC35F3* (*solute carrier family 35 member F3)*, a gene encoding a thiamin transporter, was significantly under expressed in the hooves of horses that can race barefoot repeatedly. SLC35F3 transports thiamine pyrophosphate, essential for cellular metabolism, energy production and functionality [64–67]. Thiamine deficiency severely affects cell metabolism and homeostasis [68–70].

Studies in humans have linked *SLC35F3* variants to hypertension [64, 71, 72] and thiamine supplementation has shown positive effects on reducing high blood pressure [73–75]. The precise mechanisms of thiamine's role in vasodilation and vasoconstriction are not fully understood. Reduced *SLC35F3* expression may decrease intracellular thiamine availability, leading to an opposite effect of thiamine supplementation and potentially resulting in local vasoconstriction and increased blood pressure in the hoof. Both thiamine deficiency's negative impact on metabolism as well as reduced blood flow to the hoof might seem counterintuitive. Tissue-specific regulatory elements or splice variants of *SLC35F3* might explain this observation. For example, alternative splicing could result in a transporter variant with different characteristics in hoof tissue or/and other thiamine transporters might compensate for thiamine transport in other tissues.

Reduced blood flow and its benefits to barefoot racing horses might be linked to disease prevention. Increased vasodilation in the hoof has been associated with equine laminitis, a condition characterized by increased blood flow and inflammation [76, 77]. Reduced expression *of SLC35F3* might lead to local vasoconstriction, decreasing blood flow and inflammatory response in the hoof, enhancing its durability for barefoot racing.

Overall, reduced expression of *SLC35F3* may contribute to hoof robustness by modulating local blood flow and inflammation, enabling horses to race barefoot more effectively. Interestingly, *IRX2*, another differentially expressed gene detected in our dataset, has been suggested to play a role in angiogenesis and regulates vascular endothelial growth factor A (VEGFA*)* to maintain proper vascularization in hooves [78].

### The immune system's role in supporting barefoot racing

In horses that can race barefoot repeatedly, an elevated expression of immune-related genes in hoof tissue was observed, suggesting a possible immune response associated with barefoot activity. While all horses in the study were healthy with no hoof related injuries or disease, the consistent expression of macrophage markers like CD3D, TLR6, and CCR5 points to a tissue infiltration with macrophages and chronic immune involvement, potentially aiding hoof resilience. Previous research on French trotters racing barefoot indicated inflammation in the coffin bone, which may similarly apply to our horses before sample collection [79, 80].

Macrophages, which clear necrotic cells and aid in tissue repair, might help these horses maintain and strengthen hoof tissue for future races. The concept of "inflammatory memory," where cells retain a memory of past inflammation [81–83], could explain adaptive resilience in the hooves of barefoot racing horses. Although transcripts of key transcription factors (e.g., *AP1, JUN, FOS*) linked to inflammatory memory were not detected in our dataset, related genes like *TMPRSS11*, *TNFAIP2*, *ATF3*, and *RUNX1* [82] were expressed, supporting the possibility of inflammatory memory without immediate gene expression shifts. Further studies, such as ATAC-seq analysis on hoof tissue, could clarify if trained immunity underpins hoof durability in barefoot horses.

In a 2023 study on the Baicha Iron Hoof horse, Han et al. identified 16 candidate genes potentially linked to hoof strength [24]; all but one was expressed in our dataset, yet none met our criteria for significance (S4 Table). This suggests limited overlap between genetic drivers of hoof strength in Baicha Iron Hoof and Standardbred horses. Further research will be crucial for understanding hoof resilience in barefoot racing.

## Limitations of our study

We were unable to confirm the differential expression of *TRAPPC6A* and *MT2A* via qPCR, likely due to artifacts associated with the qPCR method. Factors that can impact qPCR results include RNA quality, primer design, and PCR efficiency. All primers were tested with a four-point dilution standard curve, demonstrating comparable PCR efficiencies across genes. Although *TRAPPC6A* and *MT2A* are poorly annotated in the horse genome, Sanger sequencing confirmed that the correct genes were amplified. We used a relatively stable housekeeping gene (*bActin*), but RNA quality– potentially compromised by long-term storage and repeated freeze–thaw cycles—may have influenced the qPCR results.

*TRAPPC6A* exhibited high individual variation, possibly amplified by RNA degradation, which may have masked statistical differences. Research suggests that gene characteristics such as GC content, exon number, and gene size can also affect qPCR reliability [84, 85]. In fact, both *TRAPPC6A* and *MT2A* are relatively short genes with few exons (five in *TRAPPC6A* and two to three in *MT2A*), which can lead to inconsistent qPCR results. Additionally, specific degradation patterns, unique to certain mRNAs, could further affect outcomes [86, 87]. Therefore, RNA degradation and low expression differences likely increased individual variability, making it difficult to detect significant expression differences.

In many cases, qPCR validation may not be essential in RNA-seq studies due to the high correlation between RNA-seq and qPCR results, especially when RNA-seq is performed with adequate depth and replication, as in this study. Studies have shown RNA-seq’s reliability in accurately quantifying gene expression, making it comparable to qPCR in sensitivity and specificity [85, 88]. With technical advancements and adherence to best-practice protocols, RNA-seq alone often provides robust data, particularly in studies with rigorous statistical analysis and sufficient biological replicates [89, 90]. Moreover, validating only a few genes with qPCR may be less meaningful in genome-wide analyses, where stringent bioinformatics processing ensures result accuracy [91].

We detected a relatively low number of differentially expressed genes (DEG), and increasing the sample size in our study might have improved statistical power to detect additional DEG or to confirm existing ones. Previous studies have shown that 3–4 biological replicates per group are common practice in typical RNA-seq experiments [89, 90], while 6–8 replicates per group are recommended for studies with higher variability or smaller effect sizes [89, 90]. Our study included 7 replicates in the NB group and 11 in the B group, which meets—and even exceeds—these commonly cited recommendations.

Another factor potentially limiting statistical power is the phenotypic definition of the comparison groups. In this study, phenotyping was based on barefoot racing frequency, with both groups including horses that had raced barefoot, but to differing extents. Horses in the B group had raced barefoot at least three times within a 31-day period, whereas horses in the NB group may have raced barefoot occasionally, but never more than once within any 45-day window. For the NB group, trainers confirmed that more frequent barefoot racing was not feasible due to excessive wear on the hind hooves. This combination of objective racing data and trainer input was considered appropriate to address whether gene expression differs between horses that tolerate frequent barefoot racing and those that do not. Nonetheless, the phenotype likely represents a continuum. A stricter classification of the NB group—for instance, limiting barefoot racing to no more than once every 90 days—might have resulted in clearer group separation and improved statistical power to detect gene expression differences.

Samples were collected at different timepoints after the last race, but several components of our study design and an additional analysis aided in distinguishing between cause and consequence of barefoot racing. First, horses in both groups raced barefoot. While B horses raced barefoot at least three times within a 31-day period at least once in their career, NB horses raced barefoot occasionally, but never more than once every 45 days. This shared exposure, combined with the differences in frequency controls for effects of barefoot racing. Second, we included ‘time since last race’ as a covariate in our model and the results remained largely consistent. This suggests that the gene expression differences are not primarily driven by recent racing activity. Furthermore, for most horses, a substantial time had passed between their last race and sample collection. Taken together all these factors support the interpretation that the observed gene expression patterns are most likely associated with the underlying predisposition to tolerate frequent barefoot racing, rather than being a consequence of it.

## Conclusion

Our RNA-seq analysis has identified key genes linked to hoof strength in Standardbred trotters, offering valuable insights into their capacity to endure barefoot racing. The underlying genetic mechanisms that contribute to robust hooves appear to be complex, yet not overwhelmingly vast, functioning through the interplay of several distinct aspects of hoof biology. Our study underscores the activity of five genes—*ACCS*, *SLC35F3, TRAPPC6A*, *IRX2* and *MT2A*—in establishing the molecular foundation of hoof composition in horses suited for barefoot racing.

We found that horses capable of racing barefoot in successive competitions exhibited significantly lower expression of *ACCS*, a gene whose overexpression can lead to harmful ammonia accumulation in tissues. This suggests that reduced *ACCS* activity may enhance hoof ceramide content and tissue homeostasis, contributing to improved hoof quality. Additionally, *TRAPPC6A*, also downregulated in these horses, is involved in the production of lamellar bodies, which are essential for keratinization and maintaining the structural integrity of the hoof. The lower expression of *IRX2*, a transcription factor, indicates its potential influence on epidermal barrier function and hoof resilience through being a key regulator in keratinocyte biology and keratin production, which is crucial for cellular processes that support hoof strength and adaptability to wear and injury. Our analysis further revealed that *MT2A*, a gene involved in metal and sulfur homeostasis, was also downregulated in horses racing barefoot more frequently. This reduction may be linked to lower copper and sulfur concentrations in hoof tissue, which has implications for keratin formation and overall hoof durability. Lastly, *SLC35F3*, a thiamine transporter gene, showed reduced expression, which may lead to local vasoconstriction and decreased blood flow in the hoof, potentially enhancing hoof robustness by mitigating inflammatory responses associated with conditions like laminitis.

Together, these findings suggest that the coordinated regulation of these genes contributes to the structural and functional integrity of hooves in horses that excel in barefoot racing, offering insights into the genetic factors that underlie hoof resilience and performance in equine athletes.

## Supplementary Information

Supplementary Material 1.

Supplementary Material 2

Supplementary Material 3

## Data Availability

Data generated and analyzed in this study are included in the published article (and its supplementary files). RNA-seq data can be accessed at European Nucleotide Archive (ENA) under the accession number PRJEB83092.

## Abbreviations

ACCS: 1-aminocyclopropane-1-carboxylate synthase homolog
B: barefoot/ without shoes
DEG: differentially expressed gene (s)
ECM: ext racellular matrix
IRX2: iroquois homeobox 2
KRT: keratin
MMPs: matrix metalloproteinases
MT2A: metallothionein 2A
NB: not-barefoot/ with shoes
SLC35F3: solute carrier family 35 member F3
TRAPPC6A: trafficking protein particle complex subunit 6A
