# Supplementary material for: RNA-seq analysis identifies key genes enhancing hoof strength to withstand barefoot racing in Standardbred trotters
Source: BMC Genomics. 2025 Aug 18;26:751. doi: 10.1186/s12864-025-11814-4 (PMC12363045; doi:10.1186/s12864-025-11814-4)
Supplement: Supplementary file 1 — Supplementary Material 1. [file 12864_2025_11814_MOESM1_ESM.docx]

**S1 Table** **Primer sequences for target and housekeeping genes used to confirm the RNA-seq results.**

| **Primer name** | **Primer sequence** |
| --- | --- |
| ACTB_F | CGAGCACGATGAAGATCAAG |
| ACTB_R | GTGGACAATGAGGCCAGAAT |
| ACCS_ex14-15F | GGCTTCTTCATCTGGGCTGA |
| ACCS_ex14-15R | CCGGGCTCTTTACACTCGAA |
| SLC35F3_ex9-10F | GGTACACTGAGGACGATTCCA |
| SLC35F3_ex9-10R | TCGATGACATTCCTTGGGGA |
| TRAPPC6A_ex2-3F | GGATGAGGAGGGGGAAACTG |
| TRAPPC6A_ex2-3R | TCCAGAAGCAGATGGACAGC |
| MT2A_753.1_1F | GGAGTCGGGTGTGAATCCTG |
| MT2A_753.1_1F | ACTTGTCCGATGCCCCTTTG |
| MT2A_62.1_1F | CCCGACTGCTCCTGCGTC |
| MT2A_62.1_1R | CGGGGCAGCAGGATCTTCTT |
| IRX2_2F | GGCCCCGAGAAACAAAAG |
| IRX2_2R | TGATCCGTGAGCGAGTCC |

**S3 Table Differentially expressed genes in hooves of horses that are able to race barefoot (B) in successive races, which are associated with sulfur metabolism.** pval = *p-* value; padj = *p*-value adjusted; FC = fold change.

| **Ensemble accession number** | **Gene symbol** | **Base mean** | **pval** | **padj** | **FC** |
| --- | --- | --- | --- | --- | --- |
| ENSECAG00000021210 | *BPNT1* | 506,5 | 0,0097 | 0,5760 | -1,215 |
| ENSECAG00000008426 | *PAPSS2* | 632,3 | 0,0002 | 0,1932 | 2,905 |
| ENSECAG00000008724 | *MPST* | 3188,6 | 0,0214 | 0,7011 | -1,271 |
| ENSECAG00000018012 | *SELENBP1* | 140785,1 | 0,0357 | 0,7505 | -1,288 |
| ENSECAG00000001854 | *TST* | 1054,9 | 0,0054 | 0,4723 | -1,289 |

**S4 Table** **Genes previously discovered through sweep analysis in the Baicha Iron Hoof horse breed** (1) Values for same genes in our dataset. pval = *p-* value; padj = *p*-value adjusted; FC = fold change.

| **Ensemble accession number** | **Gene symbol** | **pval** | **padj** | **FC** | **Function** |
| --- | --- | --- | --- | --- | --- |
| ENSECAG00000010692 | *CSPG4* | 0.18 | 0.89 | 0.83 | Matrix metalloproteinase 2 activity |
| ENSECAG00000022321 | *PEAK1* | 0.06 | 0.80 | 1.35 | Matrix metalloproteinase 2 activity |
| ENSECAG00000000173 | *SEMA7A* | 0.88 | 0.99 | 1.01 | Rheumatoid arthritis |
| ENSECAG00000013581 | *CSK* | 0.03 | 0.73 | 0.85 | Rheumatoid arthritis |
| ENSECAG00000002988 | *PSTPIP1* | 0.01 | 0.66 | 1.89 | Rheumatoid arthritis |
| ENSECAG00000018191 | *GUCY1A2* | 0.78 | 0.99 | 1.08 | Rheumatoid arthritis |
| ENSECAG00000008643 | *EXPH5* | 0.44 | 0.99 | 1.15 | Inherited skin fragility |
| ENSECAG00000032285 | *EMCN* | 0.58 | 0.99 | 0.90 | Rheumatoid arthritis |
| ENSECAG00000006481 | *WWP2* | 0.56 | 0.99 | 1.03 | Plantar fibromatosis |
| ENSECAG00000024534 | *TERF2* | 0.77 | 0.99 | 1.01 | Dyskeratosis congenita |
| ENSECAG00000020408 | *PSMD7* | 0.25 | 0.94 | 0.92 | Ankylosing spondylitis |
| ENSECAG00000020378 | *NQO1* | 0.46 | 0.99 | 1.13 | Injury and inflammation |
| ENSECAG00000023748 | *NOB1* | 0.52 | 0.99 | 0.94 | Osteosarcoma |
| ENSECAG00000010266 | *NFAT5* | 0.79 | 0.99 | 1.13 | Inflammatory arthritis |
| ENSECAG00000035324 | *HAS3* | 0.28 | 0.95 | 1.58 | Hyaluronan metabolism in human keratinocytes and atopic dermatitis |

References

1. Han H, Randhawa IAS, MacHugh DE, McGivney BA, Katz LM, Dugarjaviin M, et al. Selection signatures for local and regional adaptation in Chinese Mongolian horse breeds reveal candidate genes for hoof health. BMC Genomics. 2023;24(1):35.
