# Supplementary figures and images for: RNA-seq analysis identifies key genes enhancing hoof strength to withstand barefoot racing in Standardbred trotters
Source: BMC Genomics. 2025 Aug 18;26:751. doi: 10.1186/s12864-025-11814-4 (PMC12363045; doi:10.1186/s12864-025-11814-4)

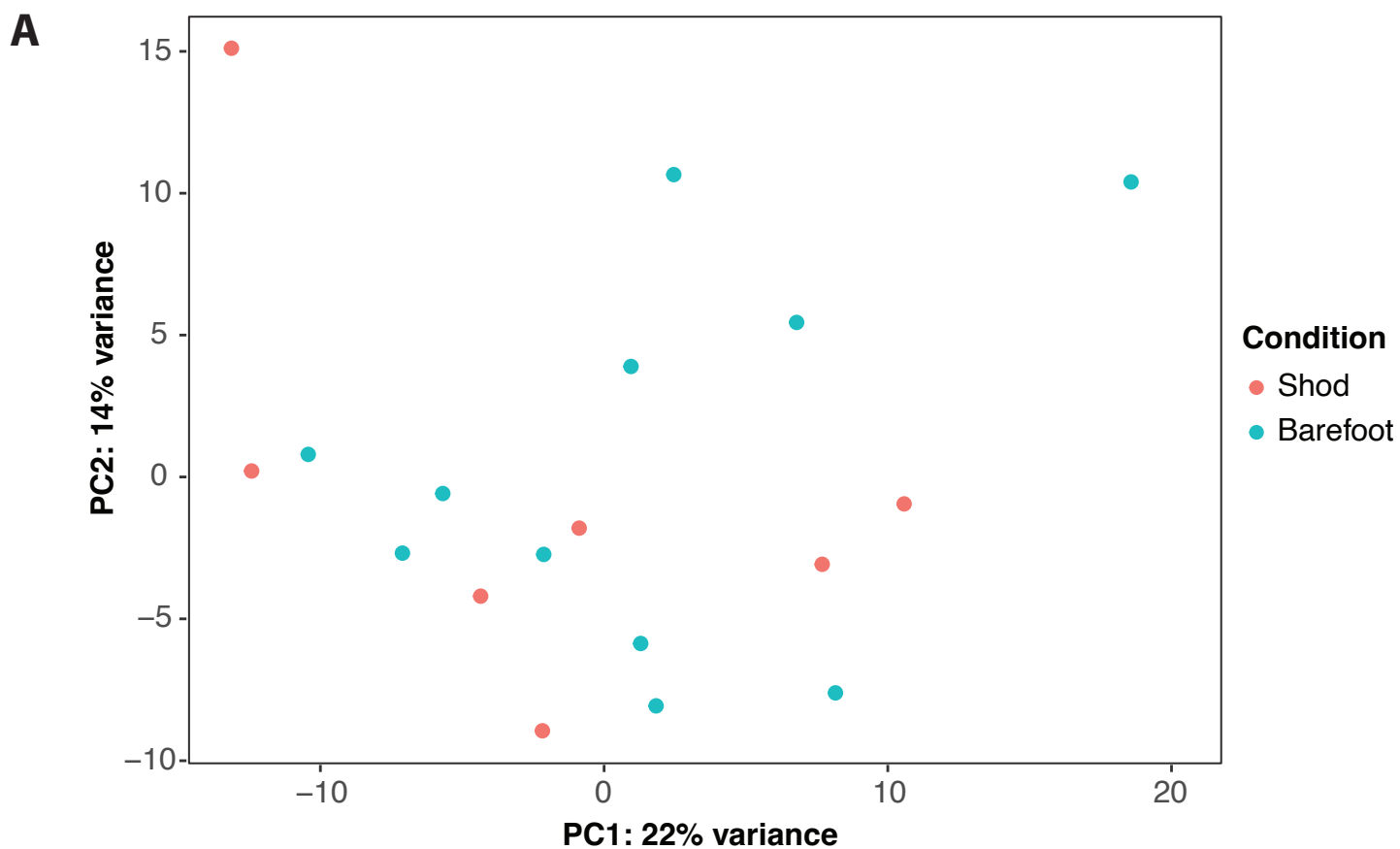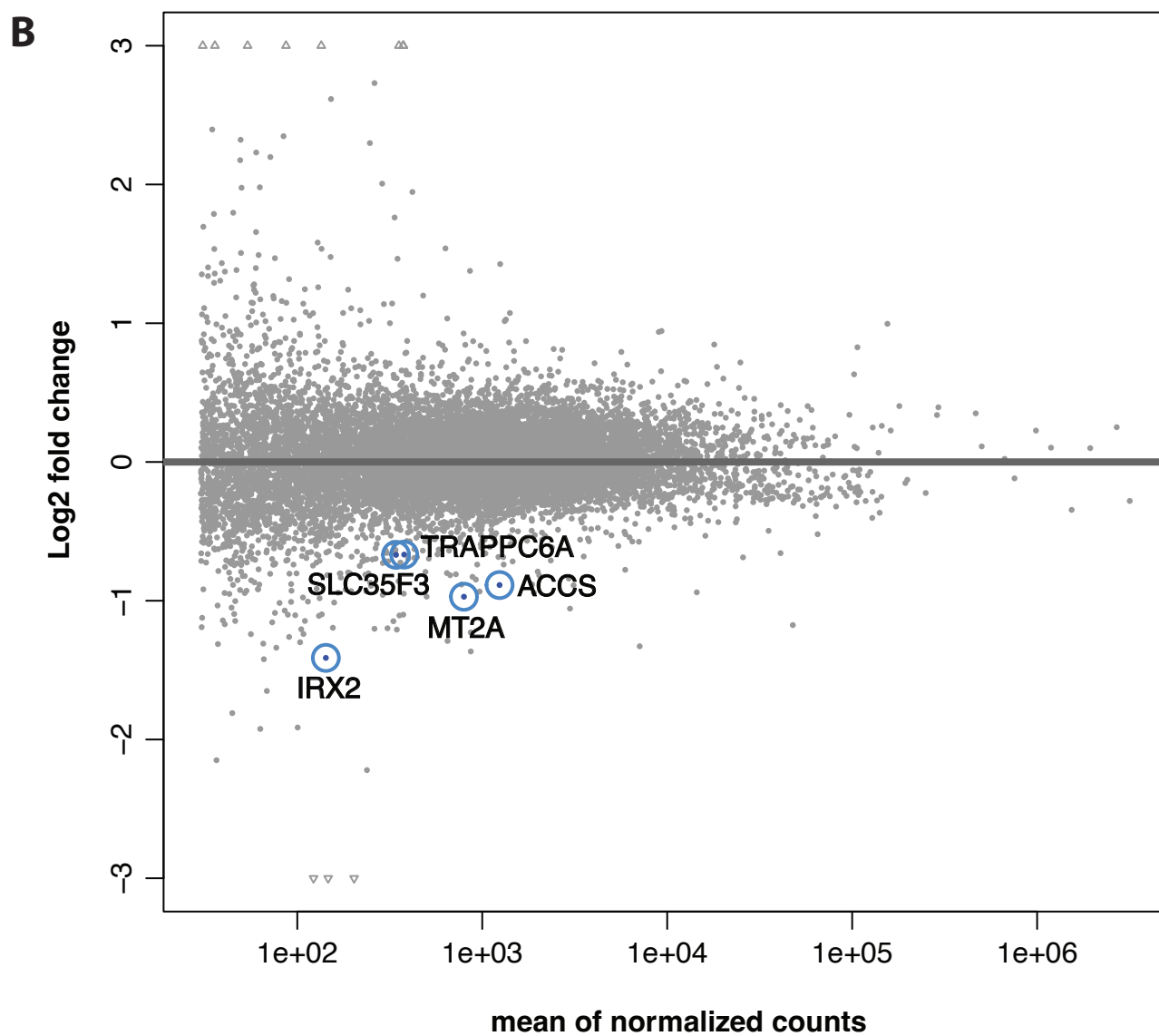

Supplement: Supplementary file 3 — Supplementary Material 3 [file 12864_2025_11814_MOESM3_ESM.pdf]
